# Outcomes and Economic Evaluation in Delayed Two-Stage Breast Reconstruction in Romania: The Influence of Radiotherapy

**DOI:** 10.3390/curroncol30020149

**Published:** 2023-02-05

**Authors:** Andrei Ludovic Porosnicu, Stefania Mihaela Riza, Ioana Antonia Stanculescu, Sorin Viorel Parasca, Cristian Radu Jecan, Ruxandra Diana Sinescu

**Affiliations:** 1 Department of Plastic Surgery and Reconstructive Microsurgery, Elias Emergency University Hospital, 011461 Bucharest, Romania; 2 Department of Plastic Surgery and Reconstructive Microsurgery, ‘Carol Davila’ University of Medicine and Pharmacy, 050474 Bucharest, Romania; 3 Clinical Emergency Hospital of Plastic Surgery and Burns of Bucharest, 010713 Bucharest, Romania; 4 Clinical Department of Plastic Surgery and Reconstructive Microsurgery, “Prof. Dr. Agrippa Ionescu” Emergency Clinical Hospital, 011356 Bucharest, Romania

**Keywords:** breast cancer, radiotherapy, breast reconstruction, alloplastic breast reconstruction, autologous breast reconstruction, costs

## Abstract

**Simple Summary:**

Following radical mastectomy in breast cancer, the trend nowadays is toward immediate breast reconstruction due to the improvement in diagnostic tools. Around 30% of the patients suffering from breast cancer will need modified radical mastectomy, and some will need postmastectomy radiotherapy (PMRT). In the presence of PMRT, the reconstructive possibilities become a subject of debate because of the increased risk of reconstructive complications. The cost of reconstruction treatment of the breast in Romania is calculated based on the diagnosis-related group, which considers the patient’s comorbidities, the type of surgical procedure, and the length of hospitalization. The main aim of this paper is to review the effects of radiotherapy on the outcome and the costs of delayed two-stage breast reconstruction in a representative cohort of patients in Bucharest, Romania.

**Abstract:**

The current paper is a retrospective cohort study conducted on sixty-seven patients who underwent two-stage breast reconstruction over a 5-year period (2015–2020). Forty-one (61.2%) patients received radiotherapy (RT group), and twenty-six (38.8%) did not (non-RT group). Data regarding patients, oncological therapies, type of reconstruction, time of hospitalization, complications, and costs were collected. The statistical analysis was performed using IBM SPSS Statistics 25. General complications were noted for 18 patients (43.9%) in the RT group and for 7 patients (26.9%) in the non-RT group. Major complications were observed only in the first group (five patients–12.2%). The mean time of hospitalization in the RT group was 14.83 days for patients with complications versus 9.83 days for those without complications and 15.5 days versus 8.63 days, respectively, in the non-RT group. The mean cost for patients without complications was 235.64 euros, whereas the cost for patients with complications was 330.24 euros (*p* = 0.001). Radiation therapy can affect the overall outcome by increasing the risk of complications and increasing costs; however, our paper shows that the association of alloplastic reconstruction in patients with radiotherapy can be performed safely and with low costs in carefully selected patients.

## 1. Introduction

It has been proven that post mastectomy radiation therapy can improve loco-regional control of the disease and overall survival in patients with breast cancer [1]. Choosing the right technique for breast reconstruction, as well as the optimal timing in patients who necessitate this kind of radiation therapy, is still a controversy among plastic surgeons [1]. Despite the recent trend of immediate breast reconstruction, delayed breast reconstruction remains a safe solution for patients with medical comorbidities, a complex oncological plan of therapy, and reluctance regarding immediate breast reconstruction [2]. Many surgeons advocate for autologous reconstruction methods in patients who need postmastectomy radiotherapy because it brings non-irradiated tissue to the affected pectoral zone. The rate of soft-tissue-related complications (wound dehiscence, skin necrosis) is lower, but the surgical techniques are more complex and can increase morbidity by prolonging the recovery time. Implant-based reconstruction has the disadvantage of using radiation-damaged tissues and the advantage of a simple and faster surgical procedure [3]. The lacking consent regarding the best breast reconstruction technique and the multiple options that can be offered to the patient are causing difficulties when making decisions. Clinical experience, patient-specific characteristics, surgeon’s preferences, and costs remain the most considered parameters in treatment option analysis [4].

The Romanian National Health System uses Diagnosis Related Groups (DRG) for the economic evaluation of health services [5]. DRG is a patient classification tool that correlates the type of patients a hospital treats (i.e., its case mix) to the costs incurred by the hospital. It calculates the charges considering the main diagnosis, secondary diagnoses, surgical procedures, age, sex, and discharge status of the patients [6]. The term ”case mix complexity” has been used to refer to an interrelated but distinct set of patient attributes, which include the severity of illness, prognosis, treatment difficulty, need for intervention, and resource requirements. The purpose of the DRG is to correlate a hospital’s case mix to resource demands and associated costs supported by the hospital. Therefore, from the DRG perspective, a hospital having a more complex case mix means that the hospital treats patients who require more hospital resources [7].

This paper aims to determine the impact of post-mastectomy radiotherapy on choosing a certain type of breast reconstruction and on predicting its outcomes and costs.

## 2. Materials and Methods

This retrospective review study was carried out on patients of two large hospitals in Bucharest, Romania: Elias Emergency University Hospital and Emergency Clinical Hospital Prof. Dr. Agrippa Ionescu, from 2015 to 2020.

Data regarding age, presence of smoking habit, oncological therapies, the time between mastectomy and reconstruction, type of breast reconstruction, duration of hospitalization, general and major complications, and costs were collected. 

All sixty-seven patients included in this study received delayed breast reconstruction following modified radical mastectomy. Postmastectomy radiation therapy (PMRT) was indicated for those patients who had four or more positive axillary lymph nodes, primary tumor size of 5 cm or more, T4 stage disease, and positive/very small free margin of resection. A total lot of patients were presented into two groups: a group that received postmastectomy radiation therapy (RT group, *n* = 41) and a group that did not (non-RT group, *n* = 26).

All patients underwent delayed breast reconstruction, using either alloplastic or autologous methods. Six patients received bilateral breast reconstruction. Alloplastic breast reconstruction was carried out by placing a submuscular tissue expander a few months after the mastectomy, when radiation therapy was complete, followed by the extraction of the expander and the insertion of a permanent implant. Full muscle coverage of the implant was performed to protect the prosthetic device and to provide a good soft-tissue envelope. Autologous breast reconstruction included soft tissue transfer methods (pediculated/free flap) with or without an implant. In the current study, we included the combined methods (flap and implant) in autologous breast reconstruction procedures.

The major complications were defined as the failure of reconstruction caused by total flap necrosis or exposure to the prosthetic device. General complications were represented by infection, hematoma, seroma, and wound dehiscence.

The costs were based on the diagnosis-related group and case mix index. For each patient, a DRG score was calculated. The final cost (FC) was generated by multiplying the DRG score with the predefined price of a previous similar case, also called weighted cases (WCs). WCs are the “virtual” cases for each DRG group resulting from analyzing similar discharged cases. The price of a weighted case (WCP) represents the reimbursement value of a virtual case, and it depends on the hospital level. The level of a hospital is given by the case mix index. The case mix index is directly proportional to the price per case.

FC = DRG score × WCP

The costs were initially expressed in RON, which is the Romanian currency (1RON = 0.20 euro) and converted into euro. The costs presented in this study did not include professional fees and the price of alloplastic materials. It is important to emphasize that the costs presented in this paper are reconstruction-related only, and do not include any reintervention, no matter what type of complications occurred afterwards.

The standard follow-up plan consisted of clinical exams that were scheduled weekly for the first month, once for the next 2 months, and then at 6 months and 1 year after breast reconstruction surgery. 

The data were analyzed using IMB SPSS Statistics 25. The *t*-test and chi-square test were used for continuous and categorical variables, respectively. Univariate analysis was performed to compare the non-RT and the RT groups. Chi-square and Fisher’s exact tests were performed to examine the differences in proportions of categorical variables between the two groups. We assume statistical significance if the type I error (p) in a test is less than 0.05. 

## 3. Results

### 3.1. Data Regarding Patients

Most patients were diagnosed with stage II (49.3%) and stage III (34.3%) invasive breast cancer. The ductal histological tumor type was found in 38 patients (56.7%). Nodal involvement was noted in 35 patients (52.2%). The median age was 47 years (range 43 to 53) at the time of reconstruction. Twenty-one patients were active smokers. Postmastectomy radiotherapy was noted in 41 patients (RT group). More data is presented in Table 1.

The interval between mastectomy and breast reconstruction in the whole group had a mean of 17.51 months, with a median of 14 months (10–22 months). Table 2 shows the correlation between the time interval and rate of complications in patients from the RT group.

### 3.2. Complications

General complications occurred in 25 patients (37.3%). Eighteen of these patients (72%) were in the RT group (*p* = 0.161). The number of complications in the RT group is presented in Table 3. Major complications occurred in five patients, all of them having received radiation therapy and three of them being active smokers (*p* = 0.336). Three patients with radiotherapy (7.3%) and major complications had expander–implant breast reconstruction.

### 3.3. Type of Breast Reconstruction

In the RT group, 26 patients (63.4%) received breast autologous reconstruction, out of which 13 patients (50%) had latissimus dorsi flap and implant, 10 patients (38.4%) underwent TRAM flap reconstruction, and 3 patients (11.5%) had DIEP flap reconstruction.

In the non-RT group, six patients (23%) were treated with autologous methods: five patients (83.3%) had latissimus dorsi flap and implant breast reconstruction, and one patient (16.6%) had DIEP flap reconstruction.

Alloplastic breast reconstruction was performed in 15 cases (36.5%) in the RT group and in 20 cases (76.9%) in the non-RT group. 

### 3.4. Hospital Length of Stay

The mean hospitalization time was 11.43 ± 6.772 days. 

In the RT group, the mean hospitalization time was 8 days (range 4–10 days) for patients who underwent alloplastic breast reconstruction and 16 days (range 8.5–18 days) for those with autologous breast reconstruction (*p* = 0.008).

### 3.5. Costs

The mean cost in the entire cohort was 260.6 euros (161.29–327.216 euros). The mean cost for patients without complications was 235.648 euros, in contrast with the cost for patients with complications, which was calculated at 330.24 euros (*p* = 0.001).

In the RT group (Table 4), the median cost for those with alloplastic breast reconstruction was 160.96 euros (range 157.2–195.8 euros), whereas the cost for those with autologous breast reconstruction was 323.3 euros (range 237.52–323.48 euros).

## 4. Discussion

The use of external radiotherapy in breast cancer treatment is more frequent nowadays [3]. There are several studies that intend to determine the effects of radiation on the soft tissues of the chest area [8]. The final effects on the skin include fibrosis and thickening. An experimental study showed that combining irradiated skin and a tissue expander implantation can lead to skin necrosis, a suboptimal surface-area gain, and poor tissue compliance [8]. Improvements to radiation techniques have made radiotherapy more precise, thus lowering collateral damage to the unaffected tissues [3]. However, the irradiated chest wall remains a challenge for the plastic surgeon, who must decide which type of breast reconstruction (alloplastic, autologous, or combinations) is the most suitable for the patient and which is the optimal time interval between the completion of the radiotherapy and the reconstruction. 

There are several papers regarding expander–implant breast reconstruction in patients who received radiotherapy [9,10]. The rate of complications ranges between 7% [9] and 52.5% [10]. In our study, the rate of all complications for patients with radiotherapy and alloplastic breast reconstruction was 12.2%, and the rate of major complications was 7.3%. Our results were comparable to those reported in the literature and showed that there is an acceptable outcome in patients with a history of radiation and delayed alloplastic breast reconstruction. 

Many authors plead for autologous breast reconstruction in patients with PMRT due to its good aesthetic results and lower complication rates [4,11,12]. Lee et al. compared the risk of reconstruction failure between autologous and alloplastic techniques in patients with radiotherapy. They showed a decrease of up to 72% in complication rates for patients with autologous reconstruction compared to prosthetic-only reconstruction [13]. In addition, a recent study showed that methods, such as fast-track surgery in autologous breast reconstruction, allow a hospital stay of only 3 days [14]. Fast-track surgery aims to reduce postoperative recovery time without increasing complications and is associated with undeniable economic benefits [15]. In our study, 31.7% of the patient with previously irradiated breast area and autologous breast reconstruction had general complications, and 4.3% had major complications, compared to 12.2% and 7.3% for implant-based reconstruction. For patients with radiation history, hospital stay was 13.8 days for those with autologous methods and 8.8 days for those with alloplastic methods. The differences between our results and the results reported in the literature may be explained by the lack of cohort homogeneity, different surgical techniques among surgeons, or poor nursing.

Cost-effectiveness analysis compares the relative costs and outcomes (effectiveness) of various medical options. In breast reconstruction, Matros et al. (2015) compared the cost-effectiveness of autologous tissue methods to implants by considering the patient’s opinion as the “effectiveness” parameter [16]. They determined that autologous methods, especially DIEP flaps, cost more than implant-based reconstruction and that the patient’s satisfaction is higher. They also suggested that autologous methods are worth choosing in patients with longer life expectancies. One limitation of their study was that it did not include patients treated with postmastectomy radiotherapy [16]. In the presence of postmastectomy radiotherapy, Razdan et al. compared the cost-effectiveness of different breast reconstruction techniques. Their study analyzed both the costs and the quality of life. In patients with locally advanced breast cancer who need radiotherapy, they suggested that immediate implant-based breast reconstruction is cost-effective (early breast mound restoration, lower costs) compared to delayed autologous methods (donor site morbidity, longer hospitalization, higher costs). However, they also advocated for autologous breast reconstruction in patients with longer life expectancies because of the long-term health-related quality of life [17]. In the current study, we compared the costs between implant-based breast reconstruction and autologous breast reconstruction, especially in those cases with postmastectomy radiotherapy. The findings were that alloplastic methods were cheaper than autologous methods (178.42 versus 297.54 euro, *p* < 0.001) in patients with radiotherapy. There are several reasons for these findings, considering the diagnosis-related group system. One can be the complexity of the surgical procedure that can influence the patient’s recovery period and time of discharge. Another can be the presence of reconstruction-related complications, which depends on the patient’s health status, compliance with the treatment, and the quality of the surgery. The presence of complications increases the DRG score by adding secondary diagnosis and the possibility of reinterventions. Similarly, both a reason and a limitation of this study is the fact that the costs of the prosthesis are covered by a special national program separately. 

In the RT group, we found that the type of reconstruction didn’t significantly influence the rate of complications, but it did significantly impact the cost burden (mean costs were 284.76 € in the RT group vs. 229.8 € for the non-RT group, *p* < 0.001). Any of the complications we noted (hemorrhage, flap necrosis, etc.) may have an impact on the cost burden. However, as a limitation of the current study, we did not consider the cost of any future possible reintervention or treatment needed for our mentioned complications.

Other limitations of this study were represented by the lack of information about the time interval between the completion of the radiotherapy and the breast reconstruction, the absence of detailed information about costs, and the unavailability of patients’ opinions on the outcome of the reconstruction.

## 5. Conclusions

It is well known that the risk of complications may increase with the complexity of the surgery, duration of the intervention (e.g., alloplastic vs. autologous reconstruction), and the need for a donor site (for autologous flap harvest).

We find it important to emphasize that, although not of statistical significance, our study showed a higher rate of complications in the RT group. In our opinion, while postmastectomy radiotherapy can be associated with a higher risk of complications, it should not be considered a contraindication for alloplastic breast reconstruction, especially in delayed breast reconstruction.

Alloplastic methods remain a good choice compared to autologous methods for selected patients with a history of radiotherapy because of the good outcome, simpler and more reliable surgical technique, fewer local or general complications, shorter hospitalization period, and lower costs.

Considering all the above, we find that the overall outcome and the cost burden when treating a postmastectomy patient that received radiation therapy can be improved by choosing an alloplastic breast reconstruction strategy.

## Figures and Tables

**Table 1:** Characteristics of RT and non-RT groups in delayed breast reconstruction.

	RT Group	Non-RT Group	*p* Value
Number of patients	41	26	
Age, mean (years)	42.27	47.64	
Active smokers (number of patients)	15	6	0.245
Autologous breast reconstruction (number of patients)	26	6	0.001 *
Alloplastic breast reconstruction (number of patients)	15	20	0.001 *
General complications (number of patients)	18	7	0.161
Major complications (number of patients)	5	0	
Average length of stay (ALOS), mean (days)	12.02	10.5	0.069
- no complications - complications	9.83 14.83	8.63 15.5	
Costs, mean (euro)	284.76	229.8	0.065
- no complications - complications	264.4 327.6	189.2 335.6	

Data represented as mean or numerical, and differences are tested with *t*-test or chi ^2^ test. * stand for statistically significant difference.

**Table 2:** Time interval between mastectomy and breast reconstruction in patients with radiotherapy.

Complications/Time Interval between Mastectomy and Breast Reconstruction in RT Group (Months)	Mean ± SD	Median (Range Interval)	*p* *
Complications (*p* = 0.385 *)	18.3 ± 6.7	20 (12–23)	0.653
No complication (*p* = 0.022 *)	18.22 ± 10.17	13.5 (10–24.25)	

* stand for statistically significant difference.

**Table 3:** Correlation between breast reconstruction type and complications in the RT group.

Complication/Breast Reconstruction Type	Alloplastic Breast Reconstruction	Autologous Breast Reconstruction	*p* *
No complications, number of patients	10	13	0.30
Complications, number of patients	5	13	

* stand for statistically significant difference.

**Table 4:** The costs of breast reconstruction in the RT group.

Type of Reconstruction	Mean ± SD (Euro)	Median (IQR) (Euro)	*p* *
Alloplastic breast reconstruction (*p* = 0.001 *)	178.42 ± 39.26	160.96 (157.2–195.8)	<0.001
Autologous breast reconstruction (*p* < 0.001 *)	297.54 ± 41.42	323.3 (237.52–323.48)	

Data represented as mean or average, and differences are tested with chi ^2^ test. * stand for statistically significant difference.

## Data Availability

The data presented in this study is available in this article.
